# Efficacy, safety, and mechanisms of herbal medicines used in the treatment of obesity: A protocol for systematic review

**DOI:** 10.1097/MD.0000000000008825

**Published:** 2018-01-05

**Authors:** Moloud Payab, Shirin Hasani-Ranjbar, Azadeh Aletaha, Nasrin Ghasemi, Mostafa Qorbani, Rasha Atlasi, Mohammad Abdollahi, Bagher Larijani

**Affiliations:** a Obesity and Eating Habits Research Center, Endocrinology and Metabolism Molecular -Cellular Sciences Institute; b Endocrinology and Metabolism Research Center, Endocrinology and Metabolism Clinical Sciences Institute, Tehran University of Medical Sciences, Tehran; c Department of Community Medicine, Alborz University of Medical Sciences, Karaj, Iran; d Chronic Diseases Research Center, Endocrinology and Metabolism Population Sciences Institute; e Evidence Based Practice Research Center, Endocrinology and Metabolism Clinical Sciences Institute; f Department of Toxicology and Pharmacology, Faculty of Pharmacy, and Pharmaceutical Sciences Research Center, Tehran University of Medical Sciences, Tehran, Iran.

**Keywords:** herbal medicines, metabolic syndrome, obesity, randomized controlled trial, systematic review

## Abstract

**Introduction:**

This systematic review protocol aims to perform a protocol for assessing the effectiveness, safety, and mechanism of herbal medicines for the treatment of obesity and metabolic syndrome. Some systematic reviews conducted earlier have evaluated the efficacy of herbal medicine to treat obesity and metabolic syndrome. Owing to the huge burden imposed by obesity in the recent years, a need is felt for conducting new systematic reviews on the topic of obesity with a focus on randomized clinical trials, we felt the need.

**Methods and analysis:**

All relevant clinical trials that examine the effectiveness of herbal medicines for the treatment of obesity and metabolic syndrome without restrictions on publication status will be applied.

Four electronic databases will be searched: PubMed, Scopus, Web of Science, and the Cochrane Central Register of Controlled Trials. The main outcome is expected to be an improvement in the body weight, body mass index, waist circumference, waist-to-hip ratio, body fat (weight or mass of visceral adipose tissue, fat mass or percent), and appetite.

Selection studies, data extraction, and risk of bias will be assessed independently by 2 authors.

**Ethics and dissemination:**

In this study, ethical approval is not required because the data that will be used are not subjects and the results will be discussed through peer-reviewed publications.

**Trial registration number:**

PROSPEROCRD42016049753.

## Abstract 2

Supplemental Digital Content is available in the text

**Key Points**

Strengths and limitations of this study:

- There are currently systematic reviews on “herbal medicine” and “obesity”. Some systematic reviews, conducted earlier, have evaluated the efficacy of herbal medicine to treat obesity and metabolic syndrome. Due to the huge burden imposed by obesity in the recent years, a need is felt for conducting new systematic reviews on the topic of obesity with a focus on randomized clinical trials. This systematic review will comprehensively assess the effectiveness, safety and mechanism of herbal medicine for the treatment of obesity.
- Two researchers will independently perform study selection, data extraction, and quality assessment to make sure that all relevant studies are included without personal biases.
- High heterogeneity in the results of various studies assessing the herbal medicine therapies may be a limitation.

## 1 Introduction

### 1.1 Description of the condition

Obesity is a medical condition in which excess body fat accumulates as a result of imbalance between energy intake and energy expenditure.^[1]^ The prevalence of obesity is pandemicaly increasing in the world.^[2,3]^ Obesity is a major public health problem that leads to many diseases, including type 2 diabetes mellitus, hypertension, dyslipidemia, coronary heart disease, some cancers, osteoarthritis, kidney disease, and sleep disorders.^[4,5]^

Researchers believe that at least ≥5%r more weight loss could be thought as a healing agent in controlling obesity. Studies have proven that weight loss of 5% to 10% can make individuals susceptible to some diseases such as type 2 diabetes mellitus; cardiovascular diseases reduce and improve other complications associated with obesity.^[6,7]^

Despite a variety of studies has been conducted in the treatment and management of this disease, its “globesity” still remains a very challenging issue.^[4]^ The increase in the accumulation of fat may be because of the increase in the absorption of fat, increased lipogenesis, reduced lipolysis, and/or any combination of these 3 processes.^[8]^

### 1.2 Description of the intervention

Besides weight loss diet, exercise, and behavioral changes, one can name the use of antiobesity drugs as a weight loss strategy among overweight and obese individuals. Currently, synthetic chemical drugs are used for the treatment of obesity that imposes high cost and bad side effects. For this reason, patients and researchers are looking for alternative treatment methods such as the use of herbal medicines and their products as safer and more effective methods for their treatment.^[9–11]^ A wide variety of herbal medicine, including their extracts or active components isolated from plants can be used for weight loss and for prevention of weight gain.

Most herbal medicines and their products have been studied in limited clinical research, and none of them has been evaluated as a definite solution for weight loss and the mechanism of action of many of them is still unknown.^[12,13]^

### 1.3 How the intervention might work

Herbal medicines and their products can cause weight loss through 5 mechanisms, namely appetite control and reducing energy intake, stimulation of thermogenesis, and increasing metabolism, inhibition of pancreatic lipase activity, and reducing of fat absorption, as well as decreasing lipogenesis and increased lipolysis.^[14,15]^

### 1.4 Why it is important to do this review

Some systematic reviews conducted earlier have evaluated the efficacy of herbal medicine to treat obesity and metabolic syndrome. Owing to the huge burden imposed by obesity in the recent years, a need is felt for conducting new systematic reviews on the topic of obesity with a focus on randomized clinical trials, we felt the need. Therefore, our systematic review aimed at updating data on potential antiobesity herbal medicine, and reviewing the scientific data, including active components, and mechanisms of action against obesity in human.

### 1.5 Objectives

The main objective of this study was to perform a systematic review to assess the study evidence for the effectiveness and safety of herbal medicines for the treatment of obesity and metabolic syndrome.

## 2 Method

### 2.1 Define Inclusion and exclusion criteria

#### 2.1.1 Types of studies

All relevant clinical trials that examine the effectiveness of herbal medicines for the treatment of obesity and metabolic syndrome without restrictions on publication status will be applied.

#### 2.1.2 Types of participants

Those studies evaluating the general adult human population (>18 years) will be included in our investigation. Those studies conducted on people who are overweight or obese (BMI >25) will be included. As well, the researchers will exclude those studies of populations restricted to specific diseases, conditions, or metabolic disorders.

#### 2.1.3 Types of interventions

Definition of herbal medicines in this study is as raw or refined products derived from plants or parts of plants (e.g., leaves, stems, buds, flowers, roots, or tubers) used for the treatment of diseases.^[9,10,14,15]^ Herbal medicine involving mixed or single herb and their active component will be included.

The comparison interventions will be included: herbal medicine compared to no treatment, herbal medicine compared to placebo, herbal medicine compared to only exercise, herbal medicine compared with synthetic medicine.

#### 2.1.4 Types of outcome measures

##### 2.1.4.1 Primary outcomes

The primary outcome is expected to be an improvement in the body weight, BMI, waist circumference, waist-to-hip ratio (WHR), body fat (weight or mass of visceral adipose tissue, fat mass or percent), and appetite.

##### 2.1.4.2 Secondary outcomes

Secondary outcome is estimated to be improvement in the levels of cholesterol (total, low-density lipoprotein [LDL], high-density lipoprotein [HDL]), blood pressure, triglyceride, and blood sugar.

### 2.2 Search methods for identification of studies

#### 2.2.1 Electronic searches

At this stage, all studies on herbal medicines and their active components used in the treatment of obesity and metabolic syndrome are searched and reviewed without no time limitation. (All studies conducted by the end of June 2016 will be considered in the systematic review).

Literature search strategies will be developed using medical subject headings and keywords.

The databases including PubMed, Scopus, Web of Science, and the Cochrane will be used. The following search terms will be used: Herbal Medicine, Herbalism, Traditional Medicine, Plants Medicinal, Phytotherapy, Folk, Remedy, Ethnopharmacology, Plant Extracts, Body Mass Index, Fat Mass, Fat Free Mass, Lean Body Mass, Body composition, Waist circumference, Waist to Hip Ratio, Waist to Height Ratio, Obesity, and Overweight.

The literature search will be limited to the English language and human subjects. The search strategy for PubMed is shown in appendix 1.

#### 2.2.2 Searching other resources

To ensure literature saturation, we will scan some reference lists of included studies or relevant reviews identified through the search.

## 3 Data collection and analysis

### 3.1 Selection of studies

After electronic searching, the records will be uploaded to a database set up by EndNote software. All studies will be independently screened based on their titles and abstracts by the review authors (MP and AA). The full text of articles potentially suitable for the review will be obtained to assess relevance based on the inclusion exclusion criteria.

**Figure 1:**
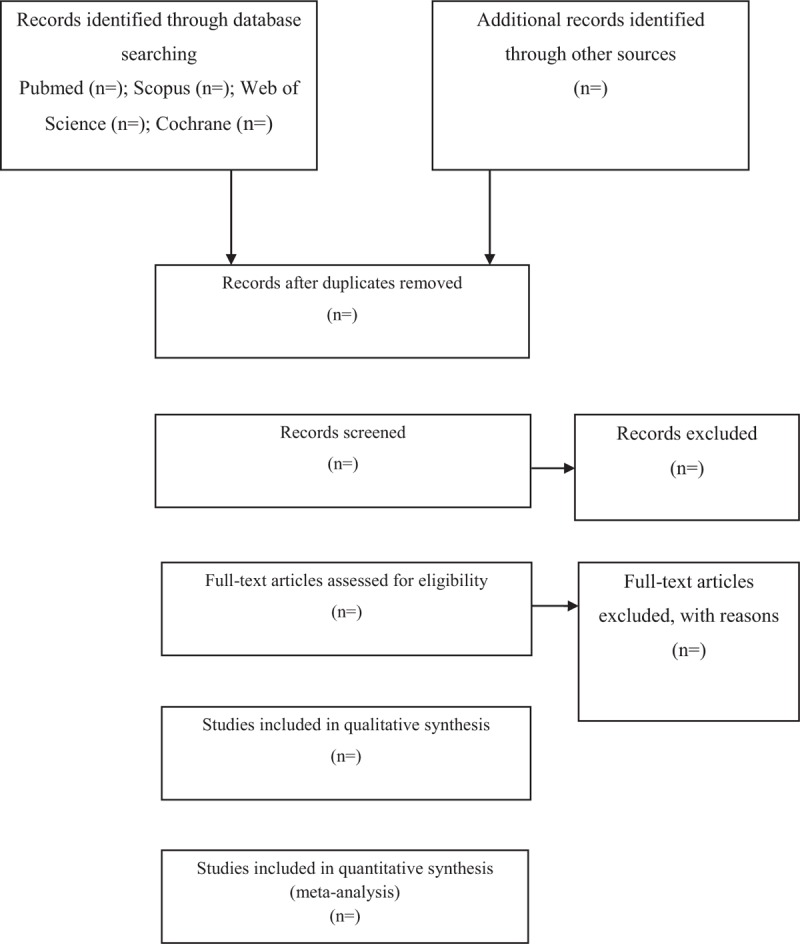
Process of the systematic review.

Any disagreements will be resolved by discussion between the 2 authors (MP and AA) and we will discuss with SHR if there are any discrepancies. The whole process of study selection is summarized in the PRISMA flow diagram (Fig. 1).

### 3.2 Data extraction and management

Data will be extracted independently from included trials by 2 authors (MP and AA) according to a predefined data extraction sheet. The extracted data will include:

1) general information (author, title, publication year, journal, location, randomization, blinding)
2) Participants (sample size, BMI range).
3) Intervention (type and name of plant, parts of plants [e.g., leaves, stems, buds, flowers, roots], active component [If noted] and duration).
4) Control (no treatment, placebo therapy).
5) Outcomes (reported outcomes, adverse events, follow-up time, and mechanism).

### 3.3 Assessment of risk of bias in included studies

The risk of bias in the included studies will be assessed independently by 2 authors (MP and AA). Two authors (MP and AA) will assess the risk of bias with CONSORT checklist^[16]^ for reporting intervention details of herbal medicines for the treatment of obesity and metabolic syndrome will be used to evaluate the risk of bias.

We will assess the following domains for risk of bias:

1. Selection bias: random sequence generation and allocation concealment.
2. Performance bias: blinding of participants, investigators, and outcome assessors.
3. Detection bias: blinding of outcome assessment.
4. Attrition bias: incomplete outcome data.
5. Reporting bias: selective outcome reporting.
6. Other bias: for example, conflicts of interest, follow-up, nonintention-to-treat or per-protocol analysis.

### 3.4 Measures of treatment effect

We will use STATA for data analysis and quantitative data synthesis. For continuous outcomes, the mean difference (MD) will be used to measure the treatment effect with 95% confidence intervals (CIs). For categorical (binary) data, we will use a risk ratio (RR) with 95% CIs for analysis.

### 3.5 Unit of analysis issues

In these trials, subjects are randomized to 2 intervention groups, and a measurement for each outcome from each subject is collected and analyzed. We will include data from parallel group-designed and cross-over designed.

### 3.6 Dealing with missing data

We will try to contact the corresponding authors of the included studies to acquire missing or insufficient data. If it is not possible to get the missing data, then only the available data will be analyzed and a sensitivity analysis will be used to determine whether the results are inconsistent.

### 3.7 Assessment of heterogeneity

Heterogeneity between studies will be assessed by the *χ*^2^-based Q test and *I*^2^ statistics. The result of *Q* test will be regarded to be statistically significant at *P* < .1. If the *I*^2^ value is <50%, the study will not be considered to have heterogeneity, but *I*^2^ value of ≥50% indicates significant statistic heterogeneity exists and we will report it and perform meta-regression analysis or a subgroup analysis to explore the possible causes.

### 3.8 Assessment of reporting biases

Publication bias will be assessed using Egger test. Funnel plots will be applied to detect potential reporting biases. Include all eligible trials, regardless of their methodological quality will be included and the interpretation of results will be done carefully based on several explanations for funnel plot asymmetry.

### 3.9 Data synthesis

If it is possible to conduct a meta-analysis, it does will be performed with Review Manager 5.3. The results of Q test show that the will be used of fixed-effects model or a random-effects model. If significant statistical heterogeneity (*P* < .1) is found, a random-effects model will be used. If no significant statistical heterogeneity is found, a fixed-effects model will be used. If heterogeneity exists, subgroup analyses or meta-regression analysis will be conducted. If any meta-analysis cannot be performed, we will summarize and explain the characteristics and results of the included studies as the systematic narrative. The results will be explained as RR for dichotomous outcomes and MD for continuous data both with 95% CIs.

### 3.10 Subgroup analysis

If significant heterogeneity is present, conducted subgroup analyses will be included.

1) Comparison between herbal medicine and no treatment
2) Comparison between herbal medicine and placebo
3) Comparison between herbal medicine and only exercise
4) Comparison between herbal medicine and synthetic medicine

### 3.11 Sensitivity analysis

Sensitivity analysis will be used to determine the effects of trial risk of bias on important outcomes. Sensitivity analysis will be conducted to verify the robustness of our primarily given assumptions of the review process. The principal decision nodes concluded the methodological quality, sample size, and the effect of missing data. The researcher will repeat the meta-analysis and low-quality studies will be excluded. The result will be compared and discussed according to the results extracted by other studies.

### 3.12 Ethics and dissemination

In this study, ethical approval is not required because the data that will be used are not subjects and the results will be discussed through peer-reviewed publications.

## 4 Discussion

A wide variety of herbal medicine, including their extracts or active compounds isolated from plants, can be used for weight loss and for prevention of weight gain. Initial analyses suggest that herbal medicine may be a cost-effective intervention in the management of obesity. The efficacy of herbal medicine for treatment of obesity and metabolic syndrome has been evaluated in previous systematic reviews. Because of the increasing number of randomized clinical trials conducted in the recent years and although some studies have enjoyed excellent methodology, the heterogeneity of their protocols prevented them to draw robust conclusions on the efficacy of herbal medicine for the treatment of obesity. Thus, a new updated, comprehensive, and objective systematic for the clinical efficacy and safety of herbal medicine for treatment obesity would be assumed as necessary to provide the needed evidence base for the future treatment recommendations. This systematic review will present an in-depth summary and the latest analysis of the current evidence for the efficacy of herbal medicine in treating obesity, which will be informative in terms of patient care and health policy.

This systematic review will aid the clinicians and can make evidence available for researchers. Patients may also enjoy potential alternative interventions. The flow chart of this systematic review is presented in Figure 1.

Our systematic review protocol was registered with the International Prospective Register of Systematic Reviews (PROSPERO) on October 18, 2016. (Registration number: CRD42016049753).

## Supplementary Material

Supplemental Digital Content
