# Supplementary material for: Efficacy, safety, and mechanisms of herbal medicines used in the treatment of obesity: A protocol for systematic review
Source: Medicine (Baltimore). 2018 Jan 5;97(1):e8825. doi: 10.1097/MD.0000000000008825 (PMC5943094; doi:10.1097/MD.0000000000008825)
Supplement: Supplemental Digital Content [file medi-97-e8825-s001.pdf]

Appendix 1.Pubmed search strategy

No	Search terms
1	("Herbal Medicine"[Mesh] ORHerbalism[TIAB] OR "Plants, Medicinal"[Mesh] OR ((Herb*[tiab] OR Plant*[tiab]) AND (Intervention* [tiab] OR treat[tiab] OR TREATMENT[TIAB] OR Therap*[tiab] OR "therapy" [Subheading] OR medicin*[tiab] OR Healing[TIAB] OR Pharmaceutical[TIAB])) OR "Phytotherapy"[Mesh] OR "Phytotherapy" [TIAB] OR "Naturopathy"[Mesh] OR Naturopath*[TIAB] OR "Drugs, Chinese Herbal"[Mesh] OR ((Chinese[TIAB] OR Drug* [TIAB]) AND Plant[TIAB]) OR "Medicine, Traditional"[Mesh] OR (Folk[TIAB] AND (Remed*[TIAB] OR medicine[TIAB])) OR (Indigenous[TIAB] AND Medicine[TIAB]) OR Ethnomedicine[TIAB] OR (Medicine[TIAB] AND Traditional[TIAB]) OR "Ethnopharmacology"[Mesh] OR "Ethnopharmacology" [TIAB] OR "Ethnobotany"[Mesh] OR "Ethnobotany"[TIAB] OR "Plant Extracts"[Mesh] OR (Plant*[TIAB] AND Extract*[TIAB]))
2	(obes*[tiab] OR Overweight[tiab] OR ((over[tiab] OR GAIN[tiab] OR HIGH[tiab] OR REDUC*[tiab] OR LOSS[TIAB]) AND (eat*[tiab] OR FOOD[TIAB] OR fat[tiab] OR fats[tiab] OR fatty[tiab])) OR "Overweight"[Mesh] OR "Body Weight"[Mesh] OR BMI[TIAB] OR (Body[TIAB] AND Mass[TIAB] AND Index[TIAB]) OR "Body Mass Index"[Mesh] OR ((Quetelet*[TIAB] OR Quetelet's[TIAB]) AND index[TIAB]) OR "Body Composition"[Mesh] OR (Body[TIAB] AND Composition[TIAB]) OR "Body Weights and Measures"[Mesh] OR (Body[TIAB] AND Composition[TIAB]) OR weight[TIAB] OR (body[TIAB] AND FAT[TIAB]) OR (Waist[TIAB] AND Circumference[TIAB]) OR (Weight[TIAB] AND length[TIAB]) OR (Waist[TIAB] AND Hips[TIAB]))
3	(("Animals"[Mesh] OR ANIMAL*[TIAB] OR RAT[TIAB] OR RATS[TIAB] OR MICE[TIAB] OR RATUS[TIAB] OR RATTUS [TIAB] OR mouse [TIAB] OR mice[TIAB] OR rabbit*[TIAB] OR sheep*[TIAB] OR murine[TIAB] OR rodent*[TIAB] OR chicken*[TIAB] OR pig[TIAB] OR pigs [TIAB] OR piglet[TIAB] OR piglets [TIAB] OR piglet[TIAB]) NOT((HUMAN*[TIAB]OR "Humans"[Mesh]) AND ("Animals"[Mesh] OR ANIMAL*[TIAB] OR RAT[TIAB] OR RATS[TIAB] OR MICE[TIAB] OR RATUS[TIAB] OR RATTUS [TIAB] OR mouse [TIAB] OR mice[TIAB] OR rabbit*[TIAB] OR sheep*[TIAB] OR murine[TIAB] OR rodent*[TIAB] OR chicken*[TIAB] OR pig[TIAB] OR pigs [TIAB] OR piglet[TIAB] OR piglets [TIAB] OR piglet[TIAB])))
4	("Clinical Trials as Topic"[Mesh] OR "Clinical Trial" [Publication Type] OR RCT[TIAB] OR NON-RANDOMIZED[TIAB] OR “NON RANDOMIZED” [TIAB] OR RANDOMIZED[TIAB] OR (CLINICAL[TIAB] AND TRIAL*[TIAB]))
5	(1 AND 2 NOT 3 AND 4)
